# Fundamentals of Quantitative Research Methods in Mental Health Nursing

**DOI:** 10.1111/jpm.13130

**Published:** 2024-10-24

**Authors:** Paul Slater

**Affiliations:** ^1^ Institute of Nursing and Health Research, Ulster University Belfast County Antrim UK

**Keywords:** mental health nursing, philosophy of research, quantitative

## 1 Introduction

These series of papers are designed to provide readers with useful information on key concepts, issues and theories when engaging in quantitative research and statistical analysis. The points identified are not exhaustive but are designed to provide the reader with key learning points, as well as direct the reader to additional reading.

The papers are designed to be incremental in learning outcomes, with the first two papers providing an overview of rudimentary principles underpinning quantitative research, data handling and quality assurance, before allowing the reader to choose which statistical techniques they need to draw upon to conduct the appropriate statistical analysis.

The statistical papers use the software package JASP 18.3.0. It is free of charge and chosen to facilitate practitioners with no access to expensive statistical software packages. Hopefully, this will promote engagement in quantitative research, service evaluation or quality improvement projects. Hopefully, we will see the fruits of these initiatives published in the Journal of Psychiatric and Mental Health Nursing.

## 2 Fundamentals of Quantitative Research Methods in Mental Health Nursing

The influence of positivism and quantitative research in psychiatric and mental health nursing research is evident across the most fundamental aspects of care provision. Be it in the establishment of clinical conditions diagnostic/classification criteria such as the DSM V or ICD 11; in the development and use of screening tools in practice such as the PHQ9 or the GAD7; or impactful policy change and the use of evidence‐based practice such as recover rates, remissions, etc. (Kutney 2006). At an international, national and regional level, we have also seen a growth of digital technology, data linkage and ethical data sharing of quantitative healthcare information to better inform service provision and provide support for evidence‐based practice. This is also the case within psychiatry and mental health nursing. Given this reliance on quantitative research, there is a clear necessity to better understand the key concepts, definitions and terms underpinning both positivism and its practical application using quantitative research.

### 2.1 Philosophical Underpinning of Positivism

Positivism is defined as ‘a philosophical system recognising only that which can be scientifically verified or which is capable of logical or mathematical proof’ (Oxford Dictionary). It is a translation of the ‘scientifically verified’ and ‘mathematical proof’ component of this definition that encapsulates quantitative research methodology and methods.

This research paradigm ‘Positivism’ has the ontological (how reality is viewed) philosophical view that reality as a single, objective reality, experienced by all people, definable, measurable and ready for change, and; epistemologically, (how the nature of knowledge is conceived) positivism seeks to describe the laws of nature in a scientifically verifiable manner, with a reliance on measurement (scientifically verifiable observations). The translation of the ontological and epistemological views of reality and how we conceive knowledge give rise to the hypothetico‐deductive model of science and from this model all quantitative research methodologies and methods emerge.

### 2.2 The Hypothetico‐Deductive Model

**FIGURE 1:**
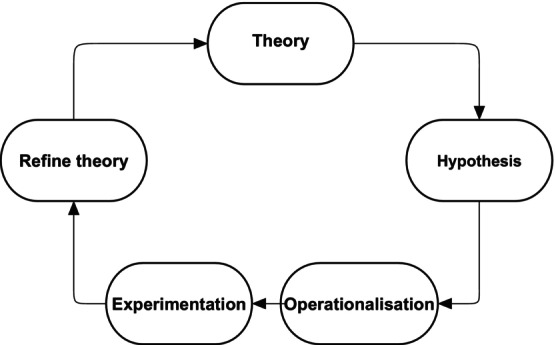
The hypothetico‐deductive model of science.

The hypothetico‐deductive model is a circular process whereby the researcher theory–hypothesis generation–operationalisation of variables–experimentation and refine theory (see Figure 1) (Park, Konge, and Artino Jr 2020).

1. Theory is derived from existing literature in the field and/or similar fields of research.
2. From this, a conceptual model may be derived with the key variables identified and the potential relationship between the variables postulated as testable hypotheses.
3. An appropriate research design, sample and setting identified to collect data.
4. Conduct experiment, collect data and test the hypotheses.
5. Refine theory based on findings.

Quantitative research methodologies and methods involve the application of the necessary tools to be able to execute the hypothetico‐deductive model in a manner that is epistemologically sound, a priori statement of intent, transparent and objective data collection, and with proven reliability and validity measures of variables.

A Working Example: In Chang et al. (2022) paper the authors used the Social Support Deterioration Model (Kaniasty and Norris 1993) to underpin the cross‐sectional survey of 300 participants with substance use disorders. Its contribution to the generation of hypothesises, instrument selection and statistical analysis.

Chang, C.W., Chang, K.C., Griffiths, M.D., Chang, C.C., Lin, C.Y. and Pakpour, A.H., 2022. The mediating role of perceived social support in the relationship between perceived stigma and depression among individuals diagnosed with substance use disorders. Journal of Psychiatric and Mental Health Nursing, 29(2), pp. 307–316.

### 2.3 Quantitative Research Methods

**FIGURE 2:**
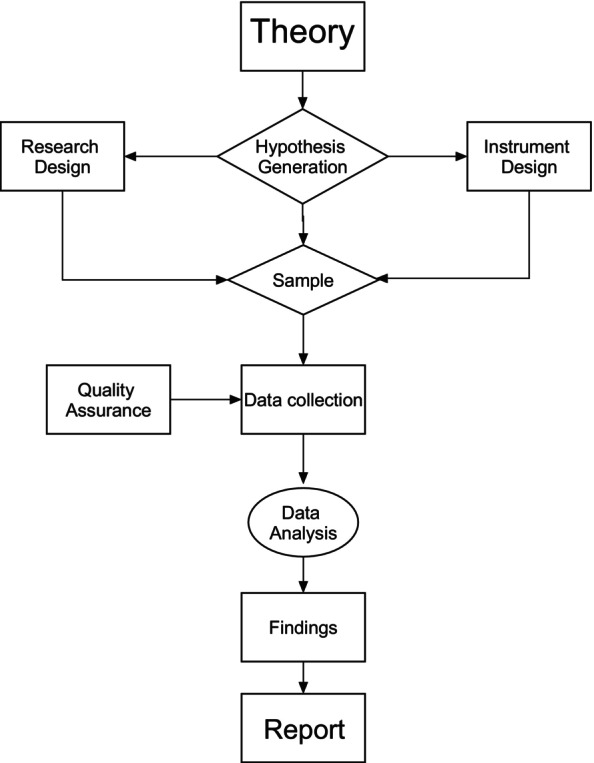
Key elements of quantitative research process.

Quantitative research can be challenging and the reliance on numerical data deters many would‐be researchers from using it as a methodology. Figure 2 shows the main elements involved in quantitative research from theory to report writing. A key feature of quantitative research is that much of the work is completed before data is collected such as hypothesis generation, research design selection, instrument selection and recruitment. The collection of data and its statistical analysis form the latter elements of the research process and focus on hypothesis testing. It is clearly an extension of the hypothetico‐deductive model and forms the template for quantitative research design.

In quantitative research, key decisions are made focusses on a number of areas in the research design process. These include the following:

- Theory and Hypothesis generation—this may emerge from a review of the literature, practice or informed by the application of a theoretical framework to a situation. a conceptual model of what we wish to examine may be constructed (see below for example) and this will also inform subsequent elements of the study such as operational definitions, instrument selection and research designs.
- Instrument selection—it is important to selection an appropriate instrument(s) to measure the intended variables otherwise the validity of the study is questionable. The instrument should be psychometrically sound and appropriate to the setting and sample that you intend to use it (based on the previous literature).
- Research design—there are four broad types of research: Randomised control trials, cohort design; case–control design; and cross‐sectional survey. Each design has varying strengths and weaknesses. Randomised control trials are regarded as the gold standard of research design. Selection of the right design is underpinned by the hypotheses you which to test.
- Data collection—is the practical process of accessing data and this includes sampling techniques and sample sizes. Care and consideration must be taken when identifying and selecting a sample and a sample size. Getting the right information from the right source, objectively and free of bias; using a reliable and valid tool is important for ensuring the quality of the findings and their generalisability to a wider audience.
- Quality Assurance and Data Analysis—the selection of the proper statistical tests to answer the research hypotheses is guided by the nature of the research design and measurement type used in the instrument. First, the quality of the data must be checked such missing data, normality of distribution, psychometrics of the instrument with the study sample. When assured of the overall quality of the data, the statistical analysis can be relatively fast and concise. The analysis of data should be focussed and not extend beyond answering the study hypotheses. In a well‐designed research study, a statistical analysis plan can be generated before data is collected (a priori).
- Write up and reporting—the researcher should be aware of the target audiences who will be interested in the study findings and disseminate accordingly. This may be in the form of reports, publications (in the Journal of Psychiatry and Mental Health Nursing hopefully!) and conference presentations to peers but it may include tweets, blogs and other forms of imaginative communication.

**HELPFUL HINT**

It is very important and beneficial to involve and methodologist and/or statistician as early as possible in a research study. They will bring valuable insight and advice to help improve the strength and generalisability of the research study findings. Waiting to the data analysis stage is often too late and many earlier mistakes cannot be corrected. Remember—statistical techniques can only correct for so many mistakes!

## 3 Conclusion

This paper provides a brief overview of the philosophical tenets of positivism and the hypothetico‐deductive model of science, and how they underpin quantitative research methodologies and methods. The aim being to produce ‘scientifically verifiable’ and ‘mathematical proof’ to examine hypotheses. Further exploration of each area identified in the paper is required to ensure that the most appropriate methodology and methods are selected to address the study objectives. This paper is the first in a series of papers to cover key areas of quantitative research methodologies and methods, intended to help promote a better understanding and increase usage of quantitative research.

## 4 Practical Example: Hypotheses Generation

First, we will look at the conceptualisation phase of a research project. Let us assume, based on our work with patients reporting high levels of anxiety and note that many report having experiences childhood traumas that are clearly anxiety‐evoking. Based on this hunch, we wish to move beyond subjective reporting, and to statistically examine the impact of childhood trauma has on development of anxiety in later life.

Quantitative research is determined by ‘a priori’ understanding of what is being measured. It is a simple conceptualisation of an idea that childhood trauma has an impact on anxiety. Unravelling and defining this model drives quantitative research.

**FIGURE 3:**
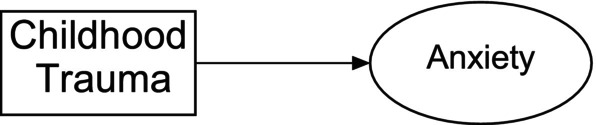
Example of a rudimentary quantitative theoretical framework.

**FIGURE 4:**
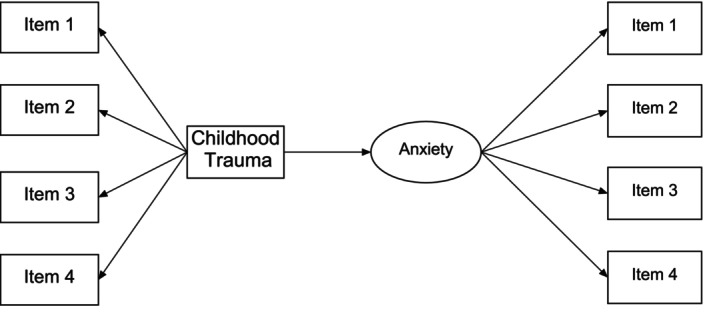
Example of theoretical framework and variable measurement.

1. There is a need to define the key terms. In this model, we are looking at the impact of childhood trauma on anxiety levels (Figure 3). For example, there are various definitions of anxiety but internationally recognised classifications of anxiety such as the DSM‐V or the ICD‐11 provide standardised definitions and allow comparison of findings with a wider audience. Similar with defining childhood trauma.
2. With this simple model, we can generate a hypothesis to test. This may be posed as a research question such as ‘what the relationship between experiencing (or not) childhood trauma on the development (or not) of anxiety is’. This hypothesis can be tested empirically.
3. With acceptable definitions, there are corresponding standardised measurement instruments. A measurement tool should align with the accepted definitions of key terms; be relevant to the population you are intending to access; and have sound psychometric properties. It may include multiple items measuring the same concept (Figure 4). The development of psychometrically sound instruments may take many years of rigorous research, and the selection of an instrument must take the psychometric evidence into consideration. Likewise, ensuring the instrument is relevant to your population—for example, there is no point asking participants with moderate/severe dementia to complete the Geriatric Depression Scale, rather a proxy tool such as the Hamilton Depression Scale by Proxy could be used.

The conceptual model identified in Figure 4 is an example of a cross‐sectional survey where participants would be asked to complete two short, 4‐item questionnaires, each examining the two constructs: childhood trauma and anxiety. In this model, childhood trauma is the independent variable and influences anxiety (the dependent variable) as indicated by the directional arrow.

Using this simple diagrammatical representation allows the identification and definition of the key variables (childhood trauma and anxiety), the relationship between the two variables as a testable hypothesis, and the identification of instruments to measure both variables. This conceptualised model is established a priori and informs and guides all further aspects of a quantitative study.

## Ethics Statement

The author has nothing to report.

## Conflicts of Interest

The author declares no conflicts of interest.

## Data Availability

The author has nothing to report.
